# An abdominal extraskeletal osteosarcoma: A case report

**DOI:** 10.3892/ol.2013.1517

**Published:** 2013-08-07

**Authors:** ZHIMING WU, XIUFENG CHU, XINGCHENG MENG, CHAOYANG XU

**Affiliations:** 1 Department of General Surgery, Shaoxing Hospital, China Medical University, Shaoxing, Zhejiang 312030, P.R. China; 2 Department of Breast and Thyroid Surgery, Shaoxing People’s Hospital, The First Affiliated Hospital of Shaoxing University, Shaoxing, Zhejiang 312000, P.R. China

**Keywords:** carcinoma, extraskeletal osteosarcoma

## Abstract

Primary abdominal extraskeletal osteosarcoma (EOS) is a rare carcinoma. The present study reports a case of a primary abdominal EOS involving the greater omentum and also presents a review of the literature on the etiology, diagnosis, differential diagnosis, pathological features, treatment and prognosis of the disease. The patient in the present study underwent laparoscopic surgery. A pathological examination revealed that the tumor tissues contained malignant and primitive spindle cells with varying amounts of neoplastic osteoid and osseous or cartilaginous tissue. The post-operative follow-up appointments were scheduled at three-month intervals for two years. The tumor recurred three months after the surgery.

## Introduction

Primary abdominal extraskeletal osteosarcoma (EOS) is rare. EOS is a malignant mesenchymal tumor, which is formed of neoplastic cells that produce bone osteoid. Since it was first described by Wilson (1) in 1941, only a limited number of studies have been published. The present study reports the case of a primary abdominal EOS involving the greater omentum, and presents a review of the literature on the etiology, diagnosis, differential diagnosis, pathological features, treatment and prognosis of the disease. Written informed consent was obtained from the patient.

## Case report

**Figure 1:**
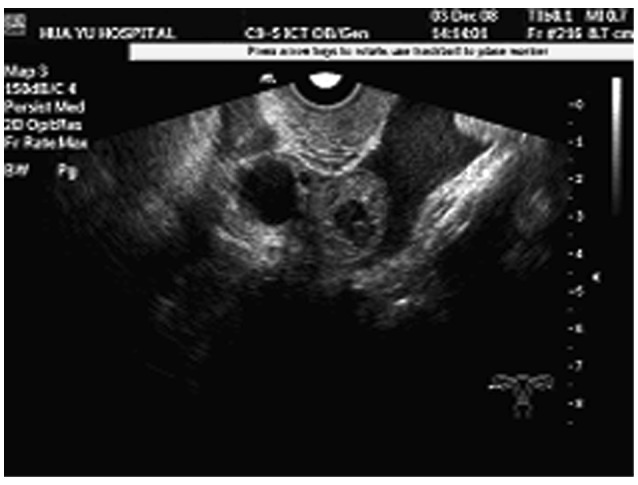
Ultrasound showing a 6.9×4.6-cm mixed echo mass in the left side of the pelvic cavity.

**Figure 2:**
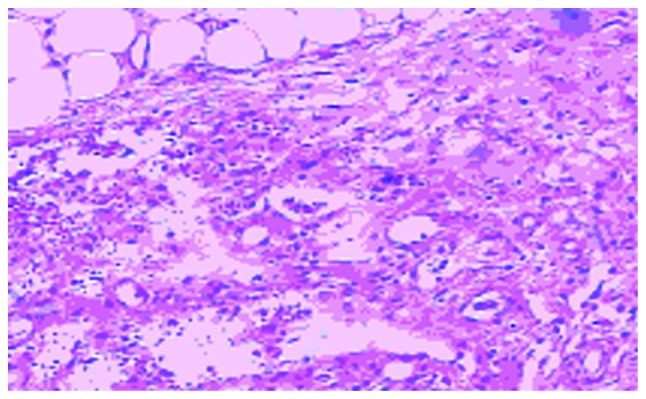
Tumor intertwined with the greater omentum (HE staining; magnification, ×100).

**Figure 3:**
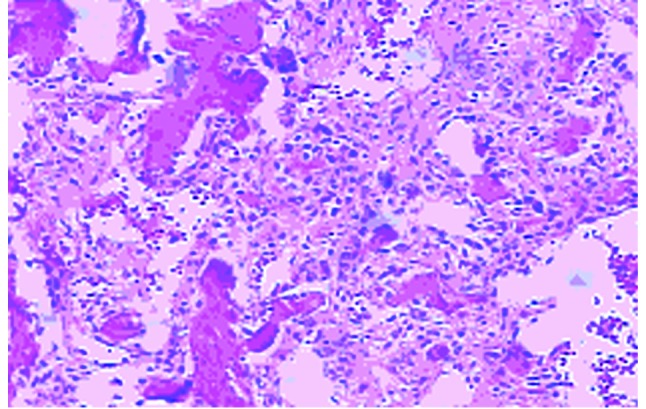
Formation of sclerotin in the tumor (HE staining; magnification, ×100).

**Figure 4:**
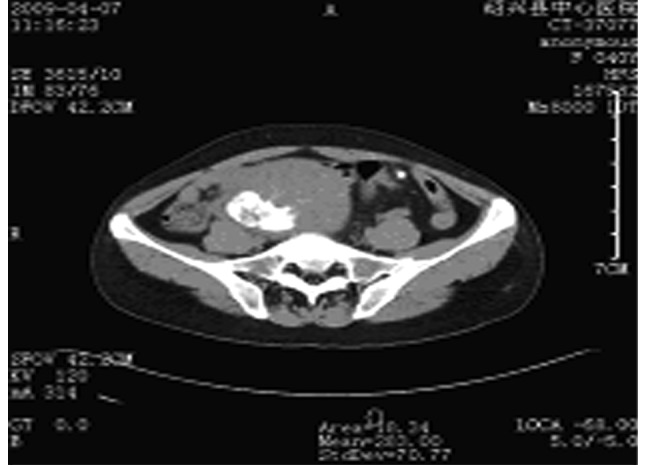
CT (computed tomography) showing a large irregular, lobulated mass in the lower abdomen and pelvis. The mass was ~13.2×13.5 cm with a large number of article sheets and an area of calcification density shadowing.

A 39-year-old female was admitted to Shaoxin Hospital on December 1, 2008 due to lower abdominal pain and discomfort for a duration of four days. Prior to admittance, the patient experienced lower abdominal pain with no obvious incentive, continuing perineum radiation pain with nausea, but without vomiting, and syncope once for ~30 sec. No chills, fever or other particular discomfort were noted and the patient had no history of surgery, trauma or radiotherapy. A physical examination revealed pain, a flat abdominal area and mild lower abdominal tenderness, but no rebound tenderness. A 6×5-cm tender mass was palpated in the left ovarian area. An ultrasound examination revealed a 6.9×4.6-cm mixed echo mass in the left side of the pelvic cavity; irregular dark areas and free dark areas were observed in the surrounding area of the mass and in the Pouch of Douglas, with cloudy areas of light (Fig. 1). The laboratory examination revealed no abnormalities in the blood, urine, stool, blood coagulation ability, biochemistry or tumor marker levels. Laparoscopic surgery was performed under intravenous anesthesia with endotracheal intubation on December 4, 2008. A 6×5-cm mass was identified on the greater omentum, located posterior to the annex and next to the left side of the uterus. The tumor was completely resected. Intraoperative frozen pathological observations revealed a malignant omentum tumor. Therefore, an omentum resection was performed. No remarkable abnormalities were identified in the abdominal cavity following a careful investigation. The microscopical examination of the paraffin sections revealed that the tumor had no capsule, but was intertwined with the greater omentum. The tumor cells were of various sizes and shapes. Dark stained nuclei and pathological karyokinesis were observed in the cells. The tumor cells were exhibiting infiltrating growth into the greater omentum (Fig. 2). Large amounts of tumor sclerotin were observed among the tumor cells (Fig. 3). Heterogeneous multi-core tumor giant cells and chondroblastoma islands were also observed. The cells were positive for vimentin and CD99 expression, but negative for cytokeratin (CK), calretinin (CR), epithelial membrane antigen (EMA), CD117, CD34, CD68, smooth muscle actin (SMA) and desmin expression by immunohistochemistry. The tumor recurred at three months post-surgery (Fig. 4). Chemotherapy was performed, including vincristine (1.4 mg/m^2^ i.v. on day 1), cyclophosphamide (750 mg/m^2^ i.v. on day 1) and doxorubicin (50 mg/m^2^ i.v on day 1) for 6 cycles; however, the patient demonstrated a poor response. The patient succumbed to systemic failure with lung metastases on June 30, 2009.

## Discussion

EOS is a rare osteosarcoma that occurs outside the bone tissue, accounting for only 1% of all soft tissue sarcomas and 4% of all osteosarcomas. EOS is a result of metaplasia of the mesenchymal tissue. The most frequent sites that are involved are the limbs, particularly the thighs and buttocks, followed by the upper extremities, retroperitoneum, gallbladder and bladder (2). EOS was first reported by Wilson in 1941 (1). The age at presentation has been recorded as between 40 and 70 years old, although EOS occurs mainly in patients that are >50 years old (3,4). The patient in the present study was 39 years old. Although the tumor was not associated with the bones and joints, the histopathological changes were consistent with that of primary osteosarcoma.

EOS has been reported to be associated with trauma, local radiotherapy, malignant fibrous tissue disease or myositis ossificans. There are two theories reported with regard to the mechanism behind EOS. The tissue residue theory suggests that the mesoblastic component forms during embryonic development and then the formation of bone and osteosarcoma occurs. The metaplasia theory suggests that muscle interstitial fibroblasts are subject to external or internal stimulation, including trauma and inflammation and metaplasia of the osteoblasts or chondrocytes, which evolves into osteosarcoma (3).

Lee *et al*(3) summarized the diagnostic criteria of EOS as follows: The tumor must arise from the soft tissue and not be attached to the bone or periosteum, it must have a uniform sarcomatous pattern, excluding mixed malignant stromal tumors, and produce an osteoid and/or cartilage matrix. X-rays will show a fairly clear soft tissue mass with punctate or flake-like calcification in the lesion, which is not connected with the surrounding bone. The diagnosis of EOS must be made using a combination of the clinical manifestation, and clinical radiographical and pathological findings. The qualitative diagnosis is confirmed by the pathological findings once the possibility of a primary bone tumor of the whole body skeletal system or a bone tumor metastasizing to the soft tissue has been excluded.

EOS tumor tissues contain malignant and primitive spindle cells with varying amounts of neoplastic osteoid and osseous or cartilaginous tissue. According to the various main components of the tumor tissues observed under a microscope, EOS may be classed as osteoblastic, chondroblastic, fibroblastic, malignant fibrous histiocytoma-like, telangiectatic and well-differentiated (5). The therapeutics of EOS are comprehensively based on surgery. According to the difference in the development, location and range of the disease, a wide excision, radical resection or simple excision may be used selectively. A previous study suggested that wide excision is effective for EOS, with an improved prognosis compared with simple excision (6). The survival rate may be improved and the rate of recurrence or metastasis may be reduced by post-operative adjuvant radiotherapy and chemotherapy. Goldstein-Jackson *et al*(6) reported that the metastasis rate was 28% for well-differentiated EOS and soft tissue sarcoma following a radical resection with adjuvant radiotherapy, while the metastasis rate was 48% for those who were not administered adjuvant radiotherapy. However, EOS is considered to be insensitive to radiotherapy or chemotherapy. Wodowski *et al*(7) reported that young adults with EOS have an improved prognosis following neoadjuvant chemotherapy.

The majority of EOSs are characterized by high invasiveness and metastasis. The prognosis is poor with high recurrence (45%) and metastasis (65%) rates. Recurrences and/or metastasis usually occur within three years following the initial diagnosis. The most common site of metastasis is the lung (>80%), followed by the regional lymph nodes, liver and heart (3,8). The median survival time for patients with EOS is 24 months and the cause-specific survival rate at five years is <25% (9). EOS may also present as a low malignancy tumor with a four to five-year survival rate following a wide excision (10,11). Bane *et al*(12) reported that mortality occurred in only one of seven patients with tumors of <5 cm in size, but in 14 of 16 patients with tumors of >5 cm, indicating that patients with tumors >5 cm have worse prognoses. In the present study, the patient presented with a tumor of >5 cm in size and was administered post-operative adjuvant chemotherapy (12). EOS recurred after three months and the patient succumbed to the disease at seven months post-surgery.
